# Online Support Program for Parents of Children With a Chronic Kidney Disease Using Intervention Mapping: A Development and Evaluation Protocol

**DOI:** 10.2196/resprot.4837

**Published:** 2016-01-13

**Authors:** Wytske W Geense, Betsie GI van Gaal, Jacqueline L Knoll, Elisabeth AM Cornelissen, Lisette Schoonhoven, Gerjo Kok

**Affiliations:** ^1^ IQ Healthcare Radboud Institute for Health Sciences Radboud University Medical Center Nijmegen Netherlands; ^2^ Department of Pediatric Nephrology Radboud University Medical Center Nijmegen Netherlands; ^3^ Clinical Academic Facility Faculty of Health Sciences University of Southampton Southampton United Kingdom; ^4^ Department of Work & Social Psychology Maastricht University Maastricht Netherlands

**Keywords:** child, chronic kidney failure, family health, health promotion, intervention mapping, parents, program development, telemedicine

## Abstract

**Background:**

The care for children with a chronic kidney disease (CKD) is complex. Parents of these children may experience high levels of stress in managing their child’s disease, potentially leading to negative effects on their child’s health outcomes. Although the experienced problems are well known, adequate (online) support for these parents is lacking.

**Objective:**

The objective of the study is to describe the systematic development of an online support program for parents of children with CKD, and how this program will be evaluated.

**Methods:**

Intervention Mapping (IM) was used for the development of the program. After conducting a needs assessment, defining program objectives, searching for theories, and selecting practical applications, the online program e-Powered Parents was developed. e-Powered Parents consist of three parts: (1) an informative part with information about CKD and treatments, (2) an interactive part where parents can communicate with other parents and health care professionals by chat, private messages, and a forum, and (3) a training platform consisting of four modules: Managing stress, Setting limits, Communication, and Coping with emotions. In a feasibility study, the potential effectiveness and effect size of e-Powered Parents will be evaluated using an explorative randomized controlled trial with parents of 120 families. The outcomes will be the child’s quality of life, parental stress and fatigue, self-efficacy in the communication with health care professionals, and family management. A process evaluation will provide insight in parents’ experiences, including their experienced level of support.

**Results:**

Study results are expected to be published in the summer of 2016.

**Conclusions:**

Although the development of e-Powered Parents using IM was time-consuming, IM has been a useful protocol. IM provided us with a systematic framework for structuring the development process. The participatory planning group was valuable as well; knowledge, experiences, and visions were shared, ensuring us that parents and health care professionals support the program.

**Trial Registration:**

Dutch Trial Registration: NTR4808; www.trialregister.nl (Archived by WebCite at http://www.webcitation.org/6cfAYHcYb)

## Introduction

### Parents Managing Their Child’s Chronic Disease

Pediatric chronic diseases affect the life of many children, as well as their families [1]. Parents have a key role in managing their child’s disease [2,3]: they have to balance their child’s health care needs against those of other family members and work commitments [2]. The increased strain on the parenting role, hospitalizations, child function impairments, and difficulties in accepting their child’s disease cause emotional problems and high levels of stress among these parents [2,4,5]. This leads to negative effects on their child’s health outcomes [1,3,5] and their quality of life [6,7].

Chronic kidney diseases (CKD) are an example of pediatric chronic diseases, posing a lot of psychological tensions on parents, as well as the children themselves [8]. Children with CKD are generally diagnosed early in life, and poor growth and development are frequently seen [9,10]. Infections, bone disease, reduced renal function, and eventually kidney failure are frequent complications among these children [11,12]. Despite interventions like renal replacement therapy or kidney transplantation, mortality remains 30 times higher compared to healthy children [11].

The care for children with CKD is complex for parents, due to complicated medication schedules, nutritional restrictions, and invasive procedures such as three times weekly hemodialysis or daily nocturnal peritoneal dialysis [12]. The parents become nurses, pharmacists, and physicians in addition to their usual parental responsibilities [12]. To help these parents cope with difficulties encountered during all stages of their child’s CKD, support and information are necessary [12]. However, interventions to assist these parents with the day-to-day management of their child’s CKD and its consequences are lacking.

### Online Support Programs for Parents

In 2008, Swallow et al [13] described the need among parents of children with CKD for continuously available, accessible, and reliable support. Online support programs are readily accessible and can lead to improvement in users’ knowledge, self-efficacy, social support, health behaviors, and clinical outcomes [14-16]. It is not remarkable that the use of online support programs for parents of chronically ill children is increasing [17-20]. Eccleston et al [1] describe in their extensively conducted Cochrane review in 2012 that many (online) support programs for parents of children with asthma, diabetes mellitus, cancer, and skin diseases improve self-efficacy, family functioning, and psychosocial well-being of these parents. However, programs for parents of children with CKD were not included.

In 2014, Swallow et al developed the first online program for parents of children with CKD stage 3-5 in the United Kingdom [21]. In their feasibility study, they concluded that the program has the potential to beneficially affect the parent’s perceived competence to manage home-based clinical care for their children [22]. In the Netherlands, we set out to develop an online support program for parents of children with CKD as well, not only focusing on children with CKD stage 3-5, but also taking children with CKD stage 1-2 into account. By developing and providing such an intervention, we aim to improve the child’s quality of life.

### Aim of the Study

The aim of this paper is to describe the systematic development of an online support program for parents of children with CKD, and how it will be tested.

## Methods

### Intervention Mapping

For the development of the online support program *“e-Powered Parents”*, Intervention Mapping (IM) was used. IM is a protocol for the systematic development of theory- and evidence-based health promotion interventions [23]. It provides health promotion planners with a framework for effective decision making for intervention planning, implementation, and evaluation. It also provides a common creative framework facilitating collaboration between researchers, health promoters, target groups, communities, intermediates, and stakeholders from different backgrounds [23]. IM has already been used for the development of online programs for preventing cyber bullying [24] or stimulating healthy nutrition and physical activity in adolescents [25,26]. Additionally, IM was used to develop support programs for parents of chronically ill children, for example, cystic fibrosis [27].

**Figure 1:**
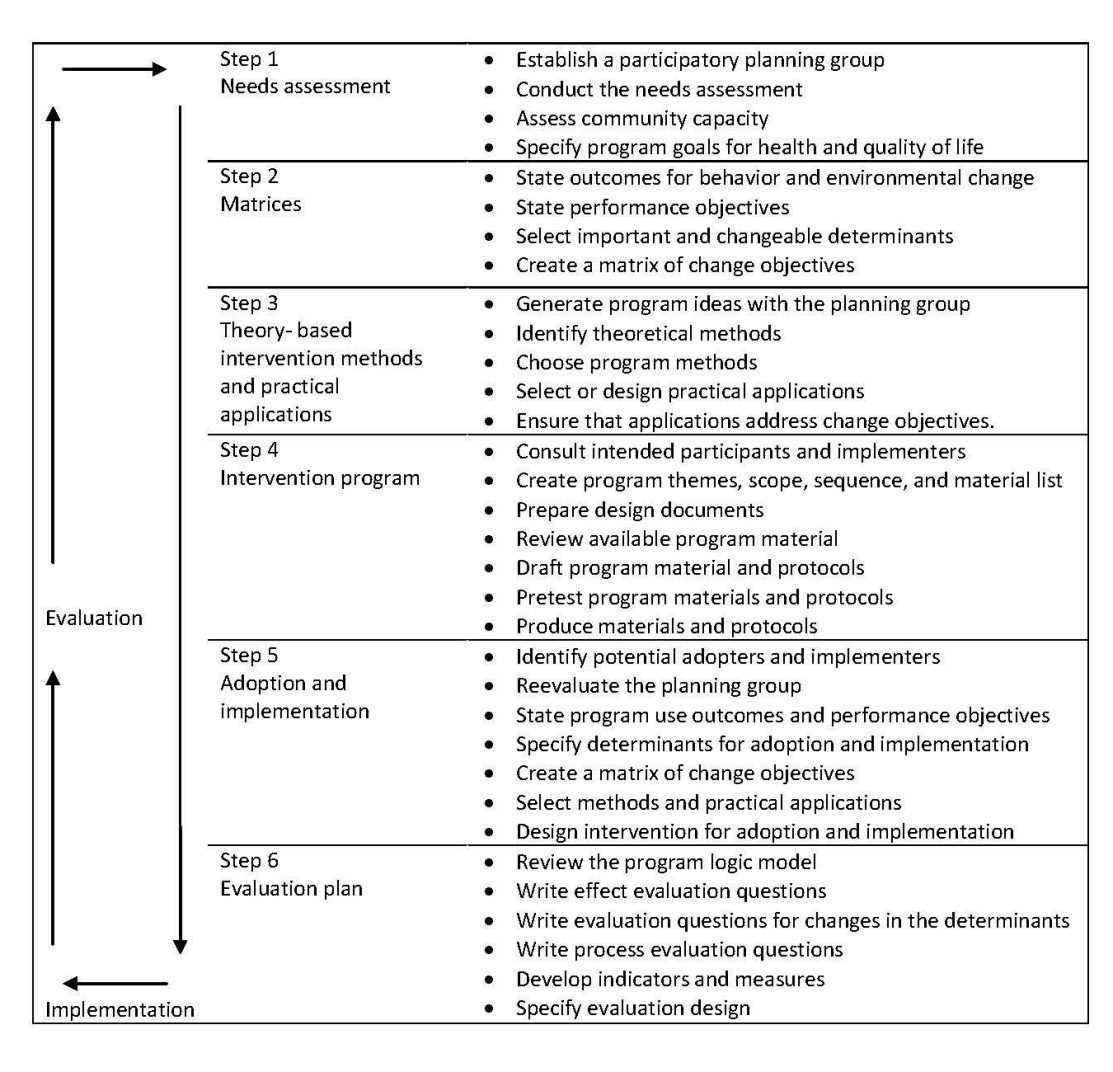
The Intervention Mapping protocol: overview of the six steps and corresponding tasks [23].

IM comprises six steps with corresponding tasks (Figure 1 shows this): (1) conducting a needs assessment; (2) identifying intervention outcomes, performance objectives, and change objectives; (3) selecting theory-based methods and practical applications; (4) developing the intervention; (5) planning for adoption and implementation; and (6) planning for evaluating the intervention. These steps will be explained and described in more detail below**.**

### Step 1 Conducting a Needs Assessment

The aim of the first step is to conduct a needs assessment to assess the health problem, its related behavior, and the environmental conditions and their associated determinants for the at-risk population [28].

First, a participatory planning group was established, consisting of important stakeholders, potential program users, and implementers. Our planning group consisted of four parents of children with CKD, six health care professionals, and two researchers (WG and BvG). The four unrelated parents were two fathers and two mothers of children with different CKDs in different stadia (for example with chronic kidney failure, on dialysis, and after transplantation). The six health care professionals were members of the pediatric nephrology team: a pediatric nephrologist (EC), a nurse practitioner (JK), a psychologist, a social worker, a dietician, and an educational worker. These professionals were involved in the daily care for children with CKD in a university hospital in the Netherlands.

The participatory planning group helped us to ensure that the intervention addresses issues important to the parents and health care professionals. In every IM step, there was a meeting with the participatory planning group to share knowledge, visions, and experiences aiming to reach consensus and to develop the intervention together.

Second, a needs assessment was conducted. The objective of a needs assessment is to get a clear understanding of the health problem, problem causing factors, and related psycho-social correlates [23].

To explore the experienced problems by parents of children with CKD, a literature and focus group study were conducted. In the literature study, PubMed, CINAHL, and PsycINFO were searched for publications between 2003-2013, on experienced problems and support needs expressed by parents in managing their child’s CKD. In the focus group study, five focus group discussions were conducted with parents of children: (1) with hereditary kidney disease (CKD stadium I); (2) with nephrotic syndrome (CKD stadium I); (3) with chronic kidney failure (CKD stadium II-IV); (4) using dialysis (CKD stadium V); and (5) after renal transplantation. All children were treated at the pediatric unit of a university hospital.

**Figure 2:**
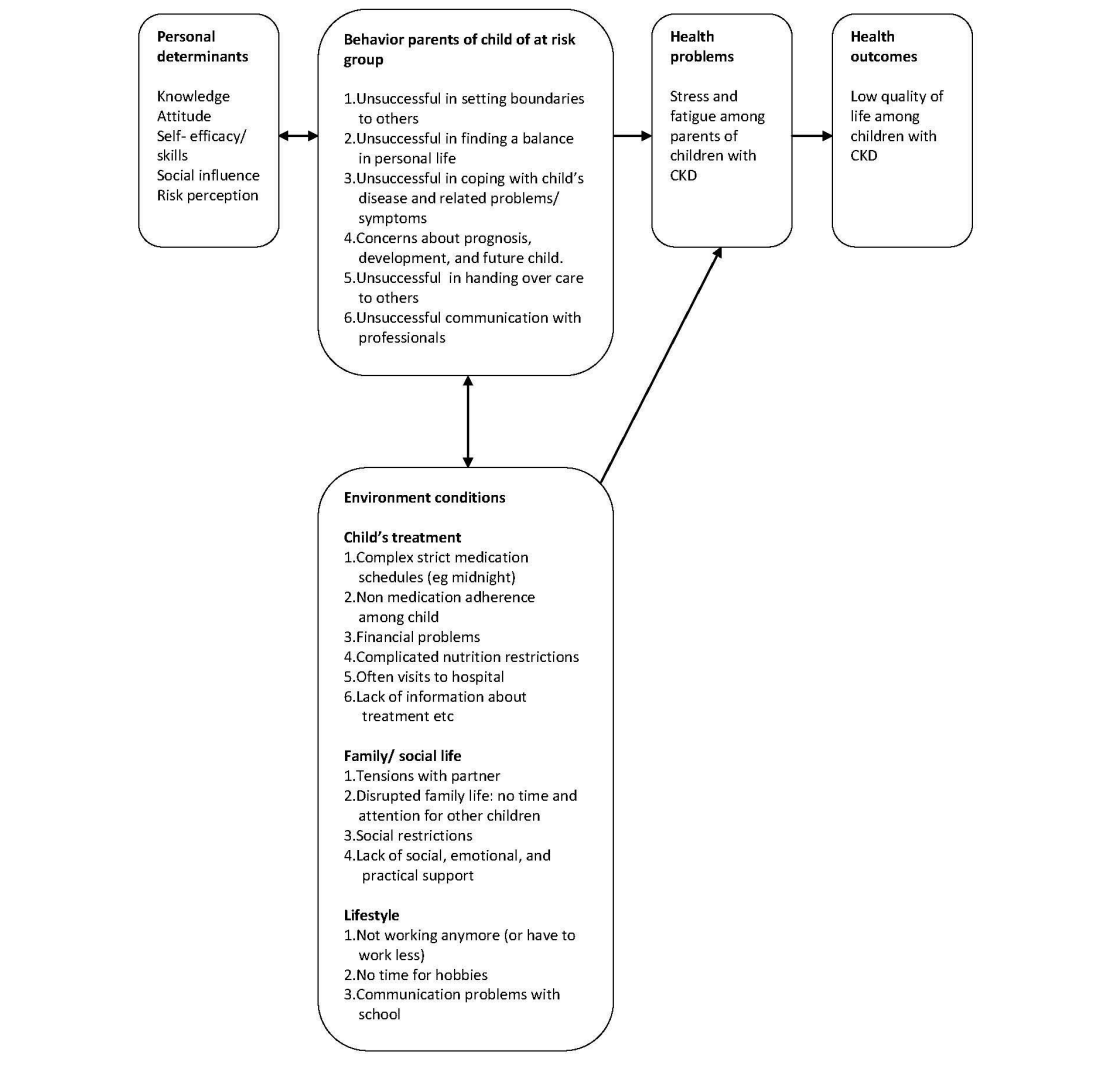
PRECEDE model [23]. Chronic kidney diseases: CKD.

The PRECEDE model by Green and Kreuter [29] is used in IM to conceptualize and guide this needs assessment, and to describe the cause of health-related and quality of life problems (Figure 2 shows this). The literature and focus group study both showed that many parents of children with CKD are tired and experience high levels of stress. Parents experience problems regarding their child’s treatment (complicated nutritional restrictions, strict medication schedules, and medication nonadherence in their child), their family and social life (tensions with partner, disrupted family life and social restrictions), and their own lifestyle (not being able to work anymore). Additionally, parents experience difficulties in setting their limits, balancing their personal life, and handing over the care for the child to others to create more time for themselves. Often, these problems are influenced by a lack of knowledge, lack of skills, and lack of (emotional, practical, and social) support from peers, family, and health care professionals. The results of the focus group study will be published in a separate article (*manuscript is under review*)*.*

The experienced problems and needs described in the needs assessment were discussed and assessed for completeness with the parents and professionals of the participatory planning group at the end of step 1.

### Step 2 Identifying Intervention Outcomes, Performance Objectives, and Change Objectives

The purpose of the second step of IM is to define, as specifically as possible, what should change in the target group and the environment in order to deal with or reduce the health-related problem.

First, program outcomes were stated, based on the needs assessment in step 1 (see Figure 2). Program outcomes are defined as the desired changes in the behavior and the environmental conditions [30]. Program outcomes regarding the behavior of the parents covered managing and coping with their own symptoms, the treatment of their child, their social activities, and their lifestyle. An example of a behavioral program outcome was, “Parents find a balance in their responsibilities as a caregiver for their child, and their own personal life”.

Second, the program outcomes were subdivided into performance objectives. Performance objectives are the required actions to accomplish the change in the behavioral and environmental outcomes [30]. What do parents have to do to find a balance in their responsibilities as a caregiver for their child, and their own personal life? Examples of performance objectives related to this outcome were, “Parents set their limits to others”, and*,* “Parents hand over their child’s care to others” (see Table 1). The participatory planning group worked together to formulate the performance objectives per program outcome.

Third, determinants were selected per performance objective (see Table 1). Determinants are those factors that are associated with the performance of behavior [30]. The focus was mainly on the determinants attitude, social influence (subjective norms), and self-efficacy (perceived behavioral control) (see Table 1) as the Theory of Planned Behavior (TPB) determined these constructs as the most important determinants of behavior [31]. The TPB has successfully been applied to many types of health behavior [23], and Internet interventions based on the TPB tend to have a large effect on behavior [32]. Besides these three determinants (attitude, social influence, and self-efficacy), knowledge and risk awareness were taken into account as well, as these are necessary prerequisites for these determinants [23].

Parents and professionals of the participatory planning group were asked to rank the determinants per performance objective, taking the importance and changeability of the determinants into account.

Fourth, change objectives were specified, by crossing the performance objectives with their determinants. Change objectives are specific goals stating what should change at an individual level [30]. For example, that what is necessary for parents to learn to hand over their child’s care to others. A change objective for the determinant “knowledge” is thereby, “Parents express the consequences of their daily care for their child and not handing over the care, on symptoms such as stress and fatigue” and a change objective for the determinant “attitude” is, “Parents express the benefits of handing over their care for their child to others”*.* For more change objectives, see Table 1.

**Table 1:** Examples of performance and change objectives of the behavioral outcome “Parents find a balance in their responsibilities as a caregiver for their child, and their own personal life.”

Performance objectives	Change objectives				
	Knowledge	Attitude	Self-efficacy	Skills	Social influence
*Parents set their limits to others*	Can mention ways to set their limits and say no	Express the benefits of setting their limits	Express their confidence in setting their limits and saying no to others, without regret	Describe step by step how they will say no to others and set their limits	Recognize that their social environment may be unaware of the burden and problems they experience
	Explain the relation between stress and fatigue and not setting their limits	Express positive attitudes toward setting limits and saying no			
*Parents hand over their child’s care to others*	Express the consequences of their daily care for their child and not handing over the care, on symptoms such as stress and fatigue	Express the benefits of handing over their care for their child to others	Express their confidence to hand over their care	Describe how they will hand over their care	Recognize that their environment really wants to support them and will take over the care
		Express positive attitude toward handing over their care for having more time for themselves			
		Accept the possibility that the caregiver can make mistakes			

### Step 3 Selecting Theory-Based Methods and Practical Applications

The aim of the third step is to review theoretical methods to effect changes in the health behavior of the individual and to change organizational and societal factors to affect the environment [33].

First, theoretical and empirical literature were searched for theory-based methods. Methods have their origins in behavioral and social science theories and are general techniques or processes for influencing changes in determinants of behavior and environmental conditions (see step 2) [33]. For example, to reach the change objective “Parents express the benefits in handing over their care for their child to others”, it is important to look at methods for changing the determinant “attitude.”

Second, for the selection of these methods, the Basic Methods for Behavioral Change described by Bartholomew et al [23] and the Coding manual for behavioral change techniques (BCTs) [34] were used. BCTs are comparable to Basic Methods for Behavior Change and based on the taxonomy of Abraham and Michie [35]. BCTs to change the determinant attitude are for example “Reevaluation of outcomes” and “Persuasive communication”.

There were two researchers (WG and BvG) who independently chose the BCTs per change objective and discussed them together until consensus was reached. The most often selected BCTs were “Providing general information” (knowledge), “Self monitoring of behavior” (awareness), “Persuasive communication” (attitude), “Reevaluation of outcomes” (attitude), “Practice” (self-efficacy), “Modeling” (self-efficacy), “Information about peer behavior” (social influence), and “Use of social support” (intention)*.* Most often, a combination of BCTs was chosen, because this is most effective in promoting behavior change [32,36,37].

Third, the chosen methods were translated into practical applications (see Table 2). A practical application is a specific technique for the practical use of theoretical methods in ways that fit with the target group and the context in which the intervention will be conducted [23]. Examples of applications for the method modeling include role playing activities or videotaped role models. In selecting the practical application, the parameters were taken into account. Parameters are conditions under which the theoretical method will be effective. A parameter of modeling, for example, is that the individual can identify him or herself with the model [23].

The final selection of the methods and practical applications was decided in a meeting with the four parents and individual meetings with the health care professionals of the participatory planning group.

It turned out that regarding the “environmental conditions” (see Figure 2), it was quite impossible to change the problems in an online program. Therefore, the decision was made to give the parents information and teach them how to cope with these environmental problems (see step 4).

**Table 2:** An overview of the determinants, used methods, and parameters in the training module “Setting limits”.

Determinant	Method (and related theory)	Parameters for use
Knowledge	Advanced organizers	Schematic representations of the content or guides to what is to be learned.
	Elaboration	Individual with high motivation and cognitive ability; messages that are personally relevant, surprising, repeated, self-pacing, not distracting, easily understandable; messages that are not too discrepant and cause anticipation of interaction.
Awareness	Self monitoring of behavior	The monitoring must be of the specific behavior (that is, not of a physiological state or health outcome). The data must be interpreted and used. The reward must be reinforcing to the individual.
	Self reevaluation/ consciousness raising	Can use feedback and confrontation; however, raising awareness must be quickly followed by increase in problem solving ability and self-efficacy.
Attitude	Persuasive communication	Messages need to be relevant and not too discrepant from the beliefs of the individual; can be stimulated by surprise and repetition. Will include arguments. For central processing of arguments they need to be new to the message receiver.
Social influence	Provide information about peer behavior	Positive expectations are available in the environment.
	Stimulate communication to mobilize social support	Combines caring trust, openness, and acceptance with support for behavioral change; assumes that positive support is available in the environment.
Self-efficacy	Planning coping responses	Identification of high risk situations and practice of coping response.
Skills	Guided practice	Sub skill demonstration, instruction, and enactment with individual feedback; requires supervision by an experienced person; some environmental changes cannot be rehearsed.
	Modeling	Attention, remembrance, self-efficacy, and skills, reinforcement of model, identification with model, coping model instead of mastery model.
	Feedback	Feedback needs to be individual, follow the behavior in time and be specific.

### Step 4 Developing the Intervention

In this fourth step, the aim is to combine the chosen applications of step 3 into a program and to develop working documents to guide the program production [38].

The components of the program were developed with the parents and health care professionals of the participatory planning group. For example, for the performance and change objectives regarding “Setting limits”, parents were asked to write a testimony regarding how they set their limits and the problems they experience. The information and materials were discussed with the social worker and psychologist.

The information technology company transformed the information into an online program, with *e-Powered Parents* as a result (the Dutch title of the online program is *“Mijn Kinderniernet”*).

*e-Powered Parents* comprises three components: (1) an informative part, (2) an interactive part, and (3) a training platform consisting of four training modules.

The informative part comprises information about different kidney diseases, treatment possibilities (including medication and nutritional restrictions), and (financial) regulations. Additional to the information, there are several folders and videos for parents and their children. This informative part focuses mainly on the environmental problems (see Figure 2).

The interactive part consists of a chat room where parents are able to chat with peers and health care professionals. Additionally, there is a forum where parents can ask questions and share their experiences, tips, and tricks with peers. Parents also have the opportunity to send private messages to other parents.

The training platform consists of four different training modules, based on the problem related behavior of the parents (see Figure 2): (1) “Managing stress”; (2) “Setting limits” (see Table 3); (3) “Communicating”; and (4) “Coping with your child’s CKD”. Every training module consists of several sessions (minimal two and maximal five). Each session starts with a welcome page, followed by a short introduction explaining what parents can expect to learn in this session, and what they have learned in the previous session. The training modules are not obligatory and can be saved temporarily. Parents themselves can select the training modules and conduct them in no particular order and as often as they want.

The program was pretested in the planning group; parents and health care professionals were asked to look closely at the information and testimonies, exercises, the ease of use, comprehensibility, and lay out of the training modules. The program was adapted using their feedback: testimonies were, for example, redrafted and spelling errors removed.

**Table 3:** Topics and sessions in the online training module “Setting limits”.

Session	Topics
1. Welcome	Short introduction in why setting limits is important for parents and what they will learn in this training module Testimony by parent why it is hard to set your limits Information how they can ask their social environment for support
2. Saying “no”, why it is important	Test how easily parents say no Information about why it is important to say no, the advantages, and why it is so difficult Testimony by parent why it is difficult Information about thoughts and their influence on saying no Exercise to write down their thoughts
3. Saying “no”, how to do?	Information about different ways to say no (sub assertive, assertive, aggressive), steps in how you can say no and what is important Exercises to say no, varying from easy to difficult and to become aware what went right and wrong Tips to discuss the exercise with their partner or friends
4. Handing over the care	Testimonies of parents why it is difficult to hand over the care for their child to others Information about why it is important to hand over their child’s care Exercise to hand over their child, for example: to become aware of the advantages of handing over care Exercise with their partner to discover which activities they find important and how they can make time for it Exercise to define their social network, to discover who can provide what kind of support Tips by health care professionals and parents to hand over care Exercise to describe what they can do when things go wrong Phone numbers of health care professionals when parents find it hard to hand over their care

### Step 5 Planning for Adoption and Implementation

The aim of the fifth step is to design a plan for the diffusion and delivery of *e-Powered Parents*. From the start of the planning process, implementation is anticipated, thereby involving parents and professionals of the participatory planning group [39].

First of all, health care professionals who are involved in the adoption and implementation of *e-Powered Parents* were identified. The key implementers, the nurse practitioner (JK) and the pediatric nephrologists (MC), were part of the participatory planning group and were involved in the development of our program. Additionally, in the weekly multidisciplinary meetings at the pediatric nephrology unit, the nurse practitioner informed the health care professionals about the progress of *e-Powered Parents*. Furthermore, a guideline with instructions was developed for parents and health care professionals for using *e-Powered Parents*.

To stimulate parents to use *e-Powered Parents*, several practical applications are planned in advance: parents who do not log in will be reminded of *e-Powered Parents* by email and during their consult with the health care professionals in the hospital. Parents who do log in will receive an email regularly with news items and updates on *e-Powered Parents*. Examples of news items are recipes for parents during birthday parties, information about yearly changes in financial regulations, or invitations for meetings. Additionally, parents will be invited to ask their questions on the forum, where health care professionals and peers are able to respond.

### Step 6 Planning for Evaluating the Intervention

The objective of the final step of IM is to design an evaluation plan specifying which information needs to be collected from the parents to have insight into the effect of the program [40].

The effect evaluation will be determined in a feasibility study, using an explorative randomized control trial (RCT) (Dutch Trial Register: NTR4808). The Medical Research Counsel guidance states that feasibility studies are essential in the development and testing of an intervention prior to a large scale evaluation [41].

The aims of the feasibility study will be to: (1) identify outcome measures most likely to capture potential benefit; (2) evaluate the potential effectiveness and effect size of *e-Powered Parents*; and (3) evaluate continued use or dropping out of *e-Powered Parents*.

Parents of 120 families, including those (1) with hereditary kidney disease (CKD stadium I); (2) with nephrotic syndrome (CKD stadium I); (3) with chronic kidney failure (CKD stadium II-IV); (4) using dialysis (CKD stadium V); and (5) after renal transplantation will be included in the explorative RCT. Stratified randomization (at family level) will be used to allocate equal numbers of parents in each of the five different categories in the control and intervention group. Parents in the control group receive the usual care, consisting of regular care and treatment for their child at a university hospital in the Netherlands. Parents in the intervention group additionally have the opportunity to use *e-Powered Parents* for six months. Both parents of a child can participate in the study, and will be randomized together into the control group or intervention group.

To explore which outcome measures are most likely to capture potential benefit, and to evaluate potential effectiveness, five outcomes were chosen: (1) The *child’s quality of life* will be measured using the Child Vulnerability Scale [42]. This proxy instrument measures the parents perceived child vulnerability, which is related to the child’s health-related quality of life [43]. (2) *Parental stress* will be measured using the Pediatric Inventory for Parents [44]; and (3) *Parental fatigue* using the Multidimensional Fatigue Inventory [45]. (4) *Self-efficacy in the communication with health care professionals* will be measured using the Perceived Efficacy in Patient-Physician Interactions [46], and (5) *Family management* using the Family Management Measure [47].

The selection of these five main outcomes is based on the PRECEDE model in the first step of IM (see Figure 2). The child’s quality of life is the “Health outcome”. Parental stress and fatigue are the “Health problems” among parents. Self-efficacy in the communication with health care professionals is a “Behavior problem”. Family management is an overall outcome, indicating the experienced difficulties by parents in managing the condition of their child.

The data will be collected, using online questionnaires, at baseline (T0) and after six months, at the end of the intervention period (T1).

For the first aim (to identify outcome measures most likely to capture potential benefit), commonly used indicators to determine the sensitivity of the outcome measures will be used; for example, floor and ceiling effects, percentage of subjects showing no change in score between T0 and T1, and the effect size of the change score [48].

For the second aim (to evaluate the potential effectiveness and effect size of *e-Powered Parents*)*,* multilevel analysis will be used to test for the posttest differences on the five outcome measures between the intervention and control group. In the multilevel analysis, there will be adjustments for families consisting of one, two, three or even four parents, and also for parents with two or more children with CKD.

The third aim (to evaluate continued participation or dropping out) is part of the process evaluation. For the process evaluation, the framework of Linnan and Steckler [49] will be used, which is endorsed by Bartholomew [23]. The key components that will be taken into account in the process evaluation are the context, reach, dose delivered, dose received, fidelity, and recruitment of the intervention [49]. Different kinds of data will be used. First, data from *e-Powered Parents* will be extracted to answer questions such as “how often did parents log in” and “which components did they visit?” Second, a short extra questionnaire will be used in the T1, to explore parents’ experiences on, for example, the ease of use of the program. Third, interviews will be conducted with parents to explore their experiences with *e-Powered Parents*. Parents who regularly visit the program will be asked about their experiences (positive and negative), their ideas to improve the program, and in which way and how the program supported parents in their stress management, setting limits, communicating with health care professionals, and coping with their child’s disease. Parents who did not log in (drop out), or only logged in once, will be asked about their reasons for not logging in and possible barriers they experienced. Additionally, interviews with health care professionals will be conducted to explore their experiences and their views on the adaptation of the program and their own role.

## Results

Study results are expected to be published in the summer of 2016.

## Discussion

### Principal Findings

The aim of this study was to describe the systematic development of an online support program for parents of children with CKD, to reduce parental stress, and thereby improve their child’s quality of life.

In the development of this online support program for parents we used IM. IM has been a useful protocol for several reasons. First, it provided us with a framework to structure the development process. It enabled us to systematically use theory, empirical evidence, and practical perspectives in the development of the intervention. Second, the formation of the multidisciplinary planning group consisting of parents, health care professionals, and researchers was valuable. Knowledge, experiences, and visions of the different members were shared. Using the participatory planning group in the developmental process, we were ensured that parents and health care professionals support this program. Third, the needs assessment in step 1 was very helpful in exploring and understanding the problems parents of a child with CKD are facing and the needs for support they have. It helped us to ensure the program addressed the needs of these parents. Fourth, although creating the matrices of the performance and change objective in step 2 was very time consuming and might become very extensive in the case of many different behaviors, the matrices were convenient in deciding which behavior the intervention should be targeting. And fifth, the review of theoretical methods in step 3 was very useful in selecting practical applications.

However, there were also challenges. First of all, the target group of this intervention is quite undifferentiated, namely parents of children with different kinds of CKDs and in different stadia. For the four parents in the participatory planning group, there were some difficulties empathizing with parents of children with different kinds of CKD and their experienced problems and needs. Moreover, in contrast to the normal route of IM, we knew already in step 1 that we wanted to develop an online program. Usually, the channel of the program is chosen in step 4. Although we are still convinced that an online program is the best channel for these parents, it might have influenced our choice for relevant methods and applications in step 3. For example, goal setting, an effective method to influence intention, was not applied in *e-Powered Parents.* Interaction between health care professionals and parents is, for example, not possible in the training modules. Subsequently, health care professionals are not able to support parents in setting their (sub) goals. Another challenge is that although the program is systematically and evidence-based developed, its success depends on its use by the parents and professionals in everyday practice.

To our knowledge, only Swallow et al [21,50] have developed an online program for parents of children with a CKD stage 3-5. There are similarities and differences between both programs. Like our program, Swallow’s program focuses on clinical care giving information and psychosocial support by using information, videos, family case studies (blogs), chat function, and a question and answer area. Both programs will also focus on managing parental stress. In our program, we also intend to change the behavior of parents in setting their limits, communicating with health care professionals, and coping with their child’s disease. Moreover, Swallow et al focus on parents of children with CKD stage 3-5, while we additionally focus on children with CKD stage 1-2. Because of these differences and similarities, it would be very interesting to compare our results after the evaluation.

Parents and health care professionals in the participatory planning group were enthusiastic about the online program and its feasibility. If the explorative trial demonstrates sufficient effectiveness of this online program, this program could be embedded in more university hospitals in the Netherlands.

### Conclusions

By applying IM, we were able to create a unique and promising online support program for parents of children with CKD in the Netherlands. Our explorative trial will indicate whether the intervention improve their child’s quality of life.

## Abbreviations

BCTs: behavioral change techniques
CKD: chronic kidney disease
IM: Intervention Mapping
RCT: randomized control trial
T0: at baseline data collection
T1: at the end of the intervention period data collection
TPB: Theory of Planned Behavior
